# Molecular and Functional Profiles of Exosomes From HPV(+) and HPV(−) Head and Neck Cancer Cell Lines

**DOI:** 10.3389/fonc.2018.00445

**Published:** 2018-10-12

**Authors:** Sonja Ludwig, Priyanka Sharma, Marie-Nicole Theodoraki, Monika Pietrowska, Saigopalakrishna S. Yerneni, Stephan Lang, Soldano Ferrone, Theresa L. Whiteside

**Affiliations:** ^1^Department of Otorhinolaryngology, Head and Neck Surgery, University of Duisburg-Essen, Essen, Germany; ^2^UPMC Hillman Cancer Center, Pittsburgh, PA, United States; ^3^Department of Otorhinolaryngology, Head and Neck Surgery, University of Ulm, Ulm, Germany; ^4^Gliwice Branch, Maria Sklodowska-Curie Institute – Oncology Center, Gliwice, Poland; ^5^Department of Biomedical Engineering, College of Engineering, Carnegie Mellon University, Pittsburgh, PA, United States; ^6^Massachussets General Hospital, Harvard Medical School, Boston, MA, United States; ^7^Departments of Pathology, Immunology and Otolaryngology, University of Pittsburgh School of Medicine, Pittsburgh, PA, United States

**Keywords:** exosomes, head and neck cancer, HPV(+) and HPV(−) tumor cells, protein profiling, immune functions

## Abstract

Exosomes produced by tumor cells have been shown to reprogram functions of human immune cells. Molecular cargos of exosomes isolated from supernatants of HPV(+) and HPV(−) head and neck cancer (HNC) cell lines or from HNC patients' plasma were compared. The exosome protein profiles resembled those of respective parent tumor cells. Only HPV(+) exosomes carried E6/E7, p16, and survivin. HPV(−) exosomes were negative for cyclin D1 and carried low p53 levels. Immunomodulatory molecules (TGF-β, FasL, OX40, OX40L, and HSP70) were carried by HPV(+) and HPV(−) exosomes. These exosomes co-incubated with human T cells induced apoptosis and suppressed T cell activation and proliferation. HPV(−) exosomes suppressed DC maturation and expression of antigen processing machinery (APM) components. In contrast, HPV(+) exosomes promoted DC maturation and did not suppress expression of APM components in mature DCs. While DCs readily internalized exosomes, T lymphocytes resisted their uptake during the initial 12 h co-culture. Thus, HPV(+) exosomes capable of sustaining DC functions may play a key role in promoting anti-tumor immune responses thereby improving outcome in patients with HPV(+) cancers.

## Introduction

Persistent infection with human papillomavirus (HPV) type 16 is a major risk factor for the development of head and neck cancers (HNCs), especially oropharyngeal squamous cell carcinoma (OPSCC) (1, 2). The HPV(+) OPSCC has clinical, histopathological, and molecular characteristics that are different from those in HPV(−) HNCs (3). HPV(+) OPSCCs occur in younger individuals without the history of smoking or alcohol abuse that is usually associated with HNC carcinogenesis (4). HPV(+) HNCs respond better to therapy, have significantly better prognosis and significantly better outcome than HPV(−) HNCs (4). Despite these differences in etiology and sensitivity to therapy, HPV(+) and HPV(−) HNCs are currently treated with the same therapeutic regimen, consisting of surgery followed by fractionated radiation, and chemotherapy (5). However, as this treatment is associated with considerable toxicity, there is a great interest in the development of more targeted and less toxic therapies for HPV(+) HNCs.

Despite intensive research efforts aimed at defining the viral, cellular and molecular mechanisms responsible for greater sensitivity to therapy and significantly better prognosis of HPV(+) HNCs, no clues have emerged so far that could explain these characteristics. It has been speculated that better prognosis of HPV(+) HNCs is due to increased activity of the host immune system conditioned by the virus, and thus able to mount a more effective anti-tumor response at the site of infection as well as systemically (6–9). However, the interplay between the HPV infection, the adaptive immune responses and the tumor microenvironment (TME) in the oropharynx rich in lymphoid tissues is not well understood. Accumulating evidence indicates that the prolonged and persistent viral infection in the local TME may drive anti-tumor immune responses, particularly after radio- and/or chemotherapy, which contribute to release of tumor-associated antigens (TAA) as well as viral antigens from dying tumor cells and potentially promote immunity (10). A better understanding of the mechanisms underlying interactions between the HPV(+) tumor cells and the host immune system is needed for the development of novel therapeutic strategies for HPV(+) OPSCC.

Tumor-derived extracellular vesicles (EVs) are emerging as an important component of the TME in human cancers (11). EVs serve as communication vehicles between the tumor and other cells in the TME and in the periphery (12). A subset of small EVs called exosomes (30–150 nm in diameter), which originate from the endocytic compartment of the parent cell and carry endocytic markers such as syntenin-1, ALIX, or TSG101, have been of special interest as potential biomarkers of disease or disease outcome (12). We have recently reported that in HNC, the protein cargo of plasma-derived exosomes informs about the tumor stage, immunosuppressive tumor profile and disease activity (13). Others have observed that in HPV(+) cancers, exosomes carry viral proteins, and genes in addition to TAA (14, 15). These data provide a rationale for focusing on exosomes in HPV(+) vs. HPV(−) HNCs as potential biomarkers discriminating between these two etiologically distinct cancers. An examination of exosomes released by HPV(+) and HPV(−) HNCs is expected to elucidate molecular signals that are delivered to recipient cells by these exosomes in the TME. The objective is to provide insights into differential capabilities of HPV(+) vs. HPV(−) exosomes to *activate* the host immune responses and thus to modulate therapeutic effects of anti-cancer immune therapies.

In this report, we use exosomes produced by HPV(+) and HPV(−) HNC cell lines as a model to study interactions of tumor-derived exosomes with human immune cells. Our data suggest that HNC-derived exosomes recapitulate molecular and viral contents of their respective HPV(+) or HPV(−) parental cells. Further, HPV(+) vs. HPV(−) exosomes differentially reprogrammed human dendritic cells (DC), but exerted similar immunoinhibitory effects on normal human T lymphocytes. The data indicate that TEX-mediated reprogramming of host immune cells is dependent on a distinct immunoregulatory cargo, which leads to subtle differential alterations in responsiveness of immune cells to antigenic stimuli. These exosome-induced alterations could explain how immune reprogramming might ultimately result in differential responses of HPV(+) vs. HPV(−) HNCs to oncological therapies.

## Materials and methods

### Tumor cell lines

Three HPV(+) cell lines (UM-SCC-2, UM-SCC-47and UPCI:SCC-90, which originated at the U. of Michigan and were isolated by Dr. Thomas Carey) and two HPV(−) cell lines (PCI-13, PCI-30) established, characterized and maintained in our laboratory (16) were cultured in 150 cm^2^ cell culture flasks and 25 ml DMEM supplemented with 1% (v/v) penicillin and streptomycin and 10% (v/v) exosome-depleted fetal bovine serum (Gibco, Fisher Scientific, Pittsburgh, PA) at 37°C and in an atmosphere of 5% CO_2_ in air. The cell expansion range varied from 40 to 80% confluency. Following 48–72 h of incubation, supernatants were collected and used for exosome isolation.

### Peripheral blood mononuclear cells

Venous blood samples were obtained from healthy volunteers. All blood specimens were centrifuged at 1,000 × g for 10 min to collect the plasma which was aliquoted and stored frozen at −80°C for exosome isolation. Heparinized blood was separated on Ficoll-Hypaque gradients (GE Healthcare Bioscience) to isolate peripheral blood mononuclear cells (PBMC). Cells were washed in medium and immediately used for experiments. All subjects donating blood specimens for this study signed an informed consent approved by the Institutional Review Board of the University of Pittsburgh (IRB #960279, IRB#0403105, and IRB #0506140). PBMCs obtained from healthy donors were used for isolation of CD4^+^ T cells by negative selection on AutoMACS (Miltenyi, San Diego, CA, USA) with a CD4^+^ T cell isolation kit (Miltenyi) as previously described by Schuler et al. (17).

### Exosome isolation from tumor cell supernatants or patients' plasma by miniSEC

Culture supernatants or freshly-thawed plasma were centrifuged at 2,000 × g for 10 min at room temperature (RT) and at 10,000 × g for 30 min at 4°C followed by filtration on 0.22 μm syringe-filters (Millipore). Pre-conditioned supernatants were concentrated from 50 to 1 mL on Vivacell 100 filter units (MWCO 100,000, Sartorius Corp, Bohemia, NY, USA). Aliquots (1 mL) of pre-conditioned plasma or concentrated supernatants were loaded on mini-SEC columns (18), and exosomes were eluted with PBS. Exosomes were collected in the void volume fraction #4 (1 mL). For some experiments, particularly for Western blots, #4 miniSEC fractions were concentrated using 100,000 MWCO Vivaspin 500 Centrifugal Concentrators (Sartorius Corp) by centrifugation at 2,000 × g for 10–15 min.

### Protein measurements

To determine protein concentration in the exosome fraction #4, Pierce BCA protein assay kit (Thermo Scientific, Rockford, lL, USA) was used according with the manufacturer's instructions.

### Transmission electron microscopy (TEM)

Freshly isolated exosomes were dispersed on 0.125% formvar/chloroform-coated copper grids and counterstained with 1% (v/v) uranyl acetate in ddH_2_O. Imaging was performed on a JEOL 1011 transmission electron microscope at the Center for Biologic Imaging at the University of Pittsburgh as previously described (18).

### Exosome size and concentration assessment by tunable resistive pulse sensing (TRPS)

Size ranges and concentrations of isolated exosome fractions were measured using TRPS as recommended by the system manufacturer Izon (Cambridge, MA, USA). Nanopores NP150 were coated with different buffers from the reagent kit supplied by Izon. Immediately before and after each experiment, calibration beads provided in the kit (200EV, at the 1:1 ratio) were tested under the same conditions used for the samples. A small volume (10 μL) of the exosome fraction #4 was diluted 1:10 in 0.03% Tween-20 in PBS and loaded on the Nanopore. The measurement conditions for the sample were as follows: NP#37266, stretch 45.6 mm, voltage 0.68 V, current 144–150 nA, and 2 pressure steps 5–12 mbar. Each particle was measured by a short drop of the current (blockade). At least 500 particles and two pressure levels were recorded for both, samples and calibration beads. The Izon software version 3.2 was used for data recording and for calculating nanoparticle size ranges and concentrations.

### Western blots

Isolated exosomes were tested for the presence of HPV(−) related and other selected exosome proteins as previously described (13). Briefly, aliquots (10 μg) of exosomes were lysed in Lane Marker Reducing Sample Buffer (Pierce, Thermo Scientific), separated on 7–15% SDS/PAGE gels and transferred onto PVDF Immobilon-P membrane (EMD Millipore) for Western blot analysis. Membranes were incubated overnight at 4°C with antibodies specific for: TSG101 (1:500, ab30871, Abcam), β-Actin (1:200, sc-47778/C4, Santa Cruz), p16/CDKN2A (1:1,000, ab108349, Abcam), Anti-Rb (1:2,000, ab181616, Abcam), Cyclin D1 (1:1,000, #2922, Cell signaling), p53 (1:200, sc-6243, Santa Cruz), SHP-2/PTPN11 (1:1,000, #3752, Cell signaling) HPV16-E6 (1:500, #251401, Abbiotec), HPV16E7 (1:500,#sc-65711, Santa Cruz); HPV16E1 (1:500, #sc53324, Santa Cruz); HPV16E2 (1:500, #ab17185, Abcam); Survivin (1:500, #ab76424, Abcam); OX40 (1:500,#sc376014, Santa Cruz); OX40 ligand (1:500. #ab156285, Abcam); HSP70 (1:500, #ab2787, Abcam). Western blot membranes were incubated in appropriate HRP-conjugated secondary antibodies (1:3,000–1:5,000, Thermo Fisher) for 1 h at room temperature (RT), and developed using ECL detection reagents (GE Healthcare Biosciences).

### Uptake of labeled exosomes by T cells or DCs

HPV(+) or HPV(−) exosomes were labeled with the PKH26 dye as previously described (19). Labeled exosomes (10 μg protein) were co-incubated with 2 x 10^5^ primary CD3+ T cells or DC in serum-free medium for 15, 60 min, 12 and 24 h at 37°C. To wash off exosomes bound to the plasma membrane, cells were pelleted and resuspended in stripping buffer (146 gNaCl, 2.5 mL acetic acid, 500 mL dd H_2_O) for 2 min, washed 3 × with PBS and fixed with freshly-prepared 1.6% (wt./vol) paraformaldehyde for 20 min at RT. Excess fixative was quenched by adding an equal volume of 1% BSA in PBS for 5 min, followed by 3 × PBS washes. Fixed cells were cytospinned onto glass slides and permeabilized with 0.1% Triton X in PBS for 1 min. To visualize F-actin and nuclei, cells were stained with Alexafluor 488-Phallodin (1:40 in 1 × PBS). Imaging was performed in Carl Zeiss LSM 800 confocal microscope.

### RNA extraction and quantitative PCR

HNSCC cell lines were pelleted, lysed in NP40 (1%) and RNA extracted using RNeasy mini kit from Qiagen following the manufacturer's instructions. For the quantitative PCR the following primers purchased from Integrated DNA Technologies were used:

HPV16 E6 F 5′-ATG CAC CAA AAG AGA ACT GC-3′
HPV16 E6 R 5′-TTA CAG CTG GGT TTC TCT AC-3′
HPV16 E7 F 5′-ATT AAA TGA CAG CTC AGA GGA-3′
HPV16 E7 R 5′-GCT TTG TAC GCA CAA CCG AAG C-3′
ß actin F 5′-TCA CCC ACA CTG TGC CCA TCT ACG A-3′
ß actin R 5′-CAG CGG AAC CGC TCA TTG CCA ATG G-3′

### Functional studies

#### CD69 down-regulation in CD4^+^ T cells

CD69 expression levels in CD4^+^ T cells were measured as previously described by Muller et al. (20). Human CD4^+^ T cells were isolated from the peripheral blood using AutoMACs and were activated with anti-CD3/anti-CD28 beads (Miltenyi) at the 1:2 beads to cell ratio in the presence of IL-2 (150 U/mL, Peprotech) for 2 h at 37°C. Exosomes obtained from the cell line supernatants (miniSEC fraction #4, 5 μg) were co-cultured with activated CD4^+^ T cells in exosome-depleted RPMI medium (Lonza) for 40 h at 37°C. Changes in CD69 expression levels on T cells were measured by flow cytometry after staining with CD69-FITC (BD Bioscience, San Jose, CA, USA) and CD4-PE (Beckman Coulter, Atlanta, GA, USA). As controls, matching-isotype control Abs, resting/non-activated T cells only (PBS) and activated T cells only (PBS) were tested in parallel.

#### CFSE-based CD4^+^ proliferation assays

CD4^+^ T cell proliferation assays were performed as previously described (13). Freshly isolated CD4^+^T cells of normal donors were labeled with 1.5 μM CFSE (Cell Trace, Thermo Scientific) in 0.1% BSA in PBS (w/v) for 10 min at 37°C, and stained cells were quenched in an equal volume of exosome-depleted FBS (Gibco). CFSE-labeled T cells (10^5^ cells/well) were activated using CD3/28 beads (at the cell to bead ratio of 1:1, T-cell activation/expansion kit, Miltenyi) for 24 h, following co-incubation with exosomes from cell lines (10 μg of fraction #4) T-cell proliferation was determined on day 4 by flow cytometry. Data were analyzed using Modfit (Verity Software House), and suppression of proliferation was compared to controls, activated T cells alone (PBS), and resting/not activated T cells as described previously (21).

#### Annexin V-based apoptosis assays with CD8^+^ Jurkat cells

CD8^+^ Jurkat cells were pre-plated (10^5^cells/well of a 96-well plate) in exosome-depleted RPMI 1640 medium for 24 h at 37°C. HNSCC cell lines were lysed using the self-made 1% NP40 lysis buffer [50 mM Tris, 150 mM sodium chloride, 0.02% (v/v) sodium azide, 1% (v/v) NP40]. Next, freshly prepared HNC cell lysates or exosomes (1–5 μg) were added to the wells and co-incubated for 24 h at 37°C. Cultures without exosomes or cell lysates and heat-killed cells (95°C for 15 min) served as controls. Apoptosis of CD8^+^ T cells was measured after 24 h co-culture using Annexin V assays (Beckman Coulter) and an Accuri flow cytometer (BD Bioscience).

#### Effects of exosomes on immature DCs (iDCs)

PBMCs were separated from whole blood of healthy donors on Ficoll-Hypaque gradients. Monocytes were isolated by positive selection with CD14+ beads (Miltenyi Biotec) using AutoMACS. Monocytes were cultured in exosome-depleted Cellgenics medium supplemented with GM-CSF (1,000 U/ml) and IL-4 (1,000 U/mL) for 6 days at 37°C. On days 0 and 3, HPV(+) or HPV(−) exosomes (10 μg/mL) or PBS as control were added to the cultures. On day 6, immature DC (iDC) were harvested and evaluated by flow cytometry for surface expression of DC-associated markers and co-stimulatory proteins (CD80, CD86, CD40) using mAbs purchased from BD Biosciences; for CD83 and HLADR with mAbs from Beckman Coulter; and for CCR7 with Abs from R&D Systems. Intracellular staining for the antigen-processing machinery (APM) components (TAP1, TAP2, LMP-7, Calreticulin, Tapasin, and ERp57) was performed using the primary mouse Abs developed and characterized as previously described (22–24). The Abs were conjugated to APC or FITC using the Lightning-Link kit (Innova Biosciences). For all antibodies, matching isotypes were used as controls.

### Analysis of functional data

Data analysis was performed using GraphPad Prism (version 6) and summarized in graphs using means and standard errors (SE). In statistical analyses, unpaired *t*-tests were used for parametric data or alternatively Mann-Whitney U tests for non-parametric data. Flow cytometry data were analyzed using VenturiOne (version 5.0, Applied Cytometry) or Kaluza (v1.5, Beckman Coulter). A *p*-value of < 0.05 was considered to be statistically significant.

The raw data supporting the conclusions of this manuscript will be made available by the authors, without undue reservation, to any qualified researcher.

## Results

### Confirmation of the HPV status of HNC cell lines

Three human HNC HPV(+) and two HPV(−) cell lines were used as a source of exosomes. Supplementary Table 1 lists the cell line designations, patients' gender, tumor sites, the HPV(−)16 and p53 status, as well as the TNM status of the tumor from which each cell line was established. All three HPV(+) cell lines were p16+ by Western blots and all three expressed mRNA for E6 and E7 (Supplementary Figure 1). The two HPV(−) cell lines were negative for p16 or for E6/E7 mRNA. The HPV(+) cell lines did not express the early antigens E1 or E2, and exosomes isolated from these cell line supernatants were negative for E1 and E2 proteins by WBs (data not shown).

### Exosome characterization

**Figure 1:**
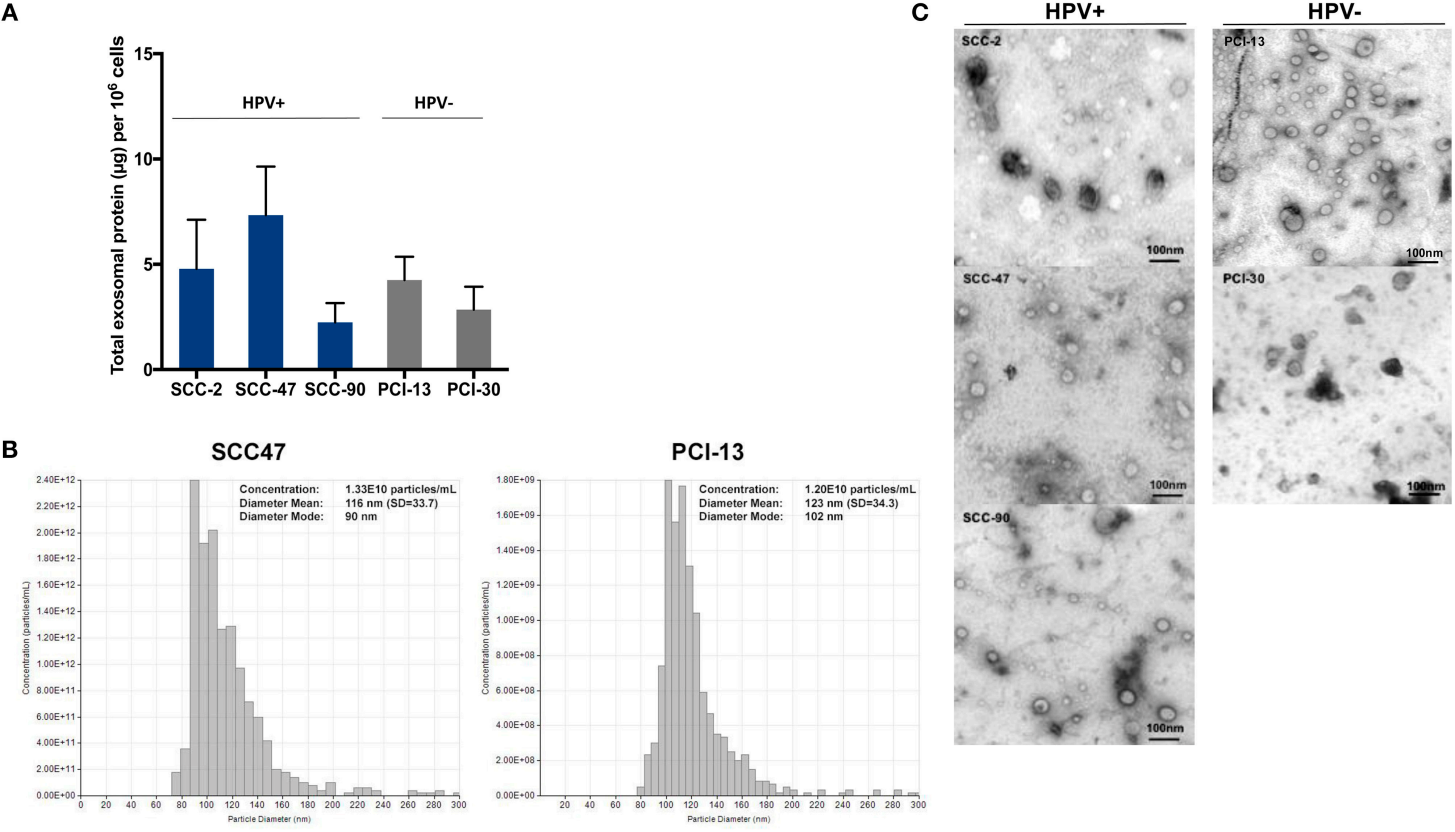
Characteristics of HPV(+) and HPV(−) exosomes. **(A)** Total protein levels in exosomes isolated from supernatants of HPV(+) and HPV(−) HNC cell lines. HNC cells were plated and expanded for 48–72 h to reach 80% confluence. Supernatants were collected pre-cleared by centrifugation, concentrated and 1 ml of the concentrate was used for exosome isolation by mini-SEC on Sepharose 2B columns. Total protein levels were measured in fractions #4. The data are means ± SD from 5 experiments with each cell line. **(B)** qNano analyses of an HPV(+) and an HPV(−) cell lines are shown. **(C)** Representative transmission electron microscopy (TEM) images of exosomes in #4 fractions of HPV(+) (SCC-2, SCC-47, SCC-90) and HPV(−) (PCI-13, PCI-30) supernatants. Representative data are from 1/3 experiments performed with each HPV(+) and HPV(−) exosomes.

**Figure 2:**
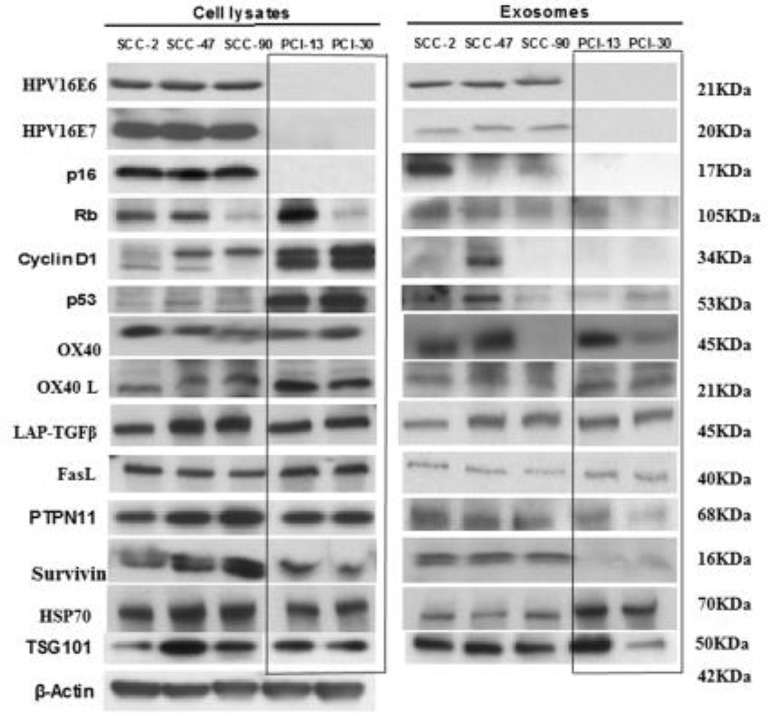
Western blot (WB) profiles of cell lysates and of matching exosomes isolated from the supernatants of the HPV(+) and HPV(−) cell lines. Each lane was loaded with 10 μg protein of the cell lysate or the corresponding exosome protein. The presence in exosomes of HPV-associated proteins (p16, Rb, Cyclin D1, p53) in HPV(+) cell lysates and exosomes and their absence in HPV(−) exosomes is evident. The blot images are grouped together, and the individual blots are shown in Supplementary Figure 2. The box separates bands for HPV(−) proteins, which are presented in opposite orientation from that shown in the original blots found in Supplementary Figure 2.

Exosomes isolated from supernatants of the cell lines by mini SEC (fraction #4) were evaluated for the total protein content (Figure 1A). Supernatants of the cell lines contained from 2 to 10 μg protein/mL per 10^6^ cultured cells. There were no significant differences between the levels of total exosome protein in HPV(+) vs. HPV(−) cells. Figure 1B shows a representative qNano profile for one HPV(+) and one HPV(−) cell line, illustrating the size and particle distribution and numbers. The qNano profiles of HPV(+) and HPV(−) exosomes were similar. Transmission electron microscopy (TEM) showed that exosomes isolated from all five HNC cell lines were also similar in size and appearance (Figure 1C). The vesicle diameter of 30–150 nm suggests that they are exosomes, and the presence of TSG101 protein in the exosome cargo seen by WBs (Figure 2) confirms their endocytic origin.

Figure 2 compares WB profiles of cell lysates with those of exosomes produced by HPV(+) or HPV(−) cells. The HPV(+) cell lines produced exosomes carrying E6/E7 proteins. These exosomes also carried Rb. Exosomes released by HPV(−) cells did not, except for PCI-13 exosomes, which were a product of cells strongly overexpressing Rb. Interestingly, p53 was strongly expressed in HPV(−) (PCI-13 and PCI-30) cells by WBs but was only minimally detectable in exosomes produced by these cells. Even more surprising was the absence of cyclin D1 in exosomes produced by PCI-13 and PC-30 cells, even though this protein was very strongly expressed in the parental tumor cells. Thus, the WB protein profiles of tumor-derived exosomes were variable and did not always correspond to those expressed by the parent cells. Survivin was carried only by HPV(+) exosomes. Notably, the T-cell inhibitory protein, PTPN11 (25) was carried predominantly by HPV(+) exosomes, although suppressive LAP-TGFβ and FasL were carried by exosomes produced by HPV(+) and HPV(−) cell lines. Co-stimulatory OX40 and OX40L and HSP70 were comparably detectable in HPV(+) and HPV(−) exosomes.

### Exosome uptake by T cells and dendritic cells (DCs)

**Figure 3:**
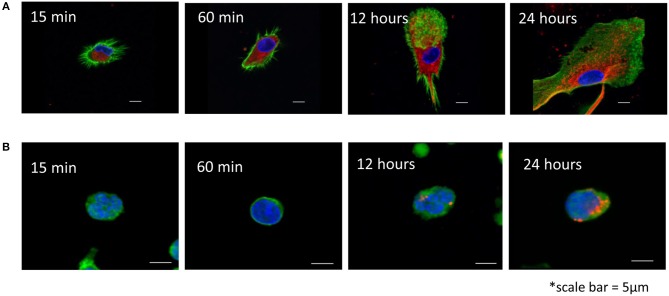
Uptake of HPV(+) exosomes by immune cells. **(A)** Human monocyte-derived iDC were co-incubated with exosomes isolated from supernatants of SCC90 cell line [HPV(+)] and labeled with a PKH26 dye (red) as described in Methods. Confocal microscopy was performed at the indicated time points. **(B)** Human primary T cells were activated as described in Methods and co-incubated with SCC90 cell line-derived exosomes for different time periods. **(A,B)** Cells were acid washed to remove surface-bound exosomes. Nuclei are blue, exosomes are red and F-actin is green. Representative images are from 1/3 experiments performed with the recipient cells obtained from different donors. In an experiment performed with HPV(−) exosomes, uptake of labeled vesicles into iDC or T cells was not different from that in images shown for HPV(+) exosomes.

Exosomes produced by HPV(+) or HPV(−) cells were labeled with the PKH26 dye and co-incubated with human activated T cells or DCs for various time periods (15 min to 24 h). Uptake of labeled exosomes by recipient cells was evaluated by confocal microscopy performed after the cells were washed with acid buffer to remove vesicles bound to the cell surface. Figure 3 shows that while DCs rapidly internalized labeled exosomes in the first 15–30 min of coincubation, T cells were reluctant to internalize exosomes, so that their uptake was evident only after 24 h of coincubation. No differences in exosome uptake between HPV(+) vs. HPV(−) exosomes was observed.

### Functions of HPV(+) and HPV(−) exosomes produced by cell lines

**Figure 4:**
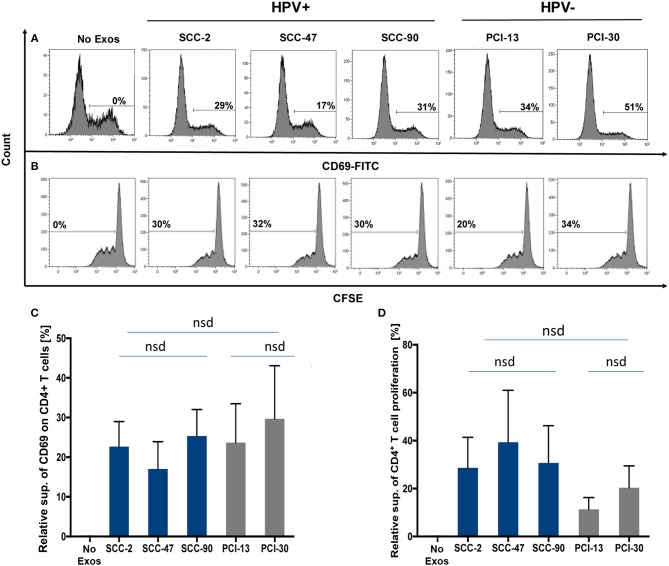
Co-incubation of HPV(+) or HPV(−) exosomes with primary lymphocyte subpopulations. **(A)** Representative flow cytometry data for exosome-mediated inhibition of CD69-expression levels on the surface of human activated CD4^+^ T cells (top row). **(B)** Representative data for suppression of CFSE-labeled CD4^+^ T cell proliferation (bottom row). **(C)** The data for relative suppression of CD69 expression levels on T cells, and in **(D)** of T cell proliferation by exosomes isolated from HPV(+) and HPV(−) exosomes are compared. The data in **(C,D)** are means ± SD for 3 experiments performed with exosomes isolated from supernatants of each of the 5 HNC cell lines studied. nsd, no significant difference.

Exosomes produced by HPV(+) or HPV(−) cells were co-incubated with normal human CD4^+^ T cells isolated from PBMC to compare effects of these exosomes on cellular activation, proliferation or apoptosis of recipient T cells. Exosome-mediated suppression of CD69 expression levels on activated CD4^+^ T cells (Figure 4A) or suppression of CD4^+^ T-cell proliferation by these exosomes (Figure 4B) were not significantly different in CD4^+^ T cells co-incubated with HPV(+) vs. HPV(−) exosomes Figure 4.

**Figure 5:**
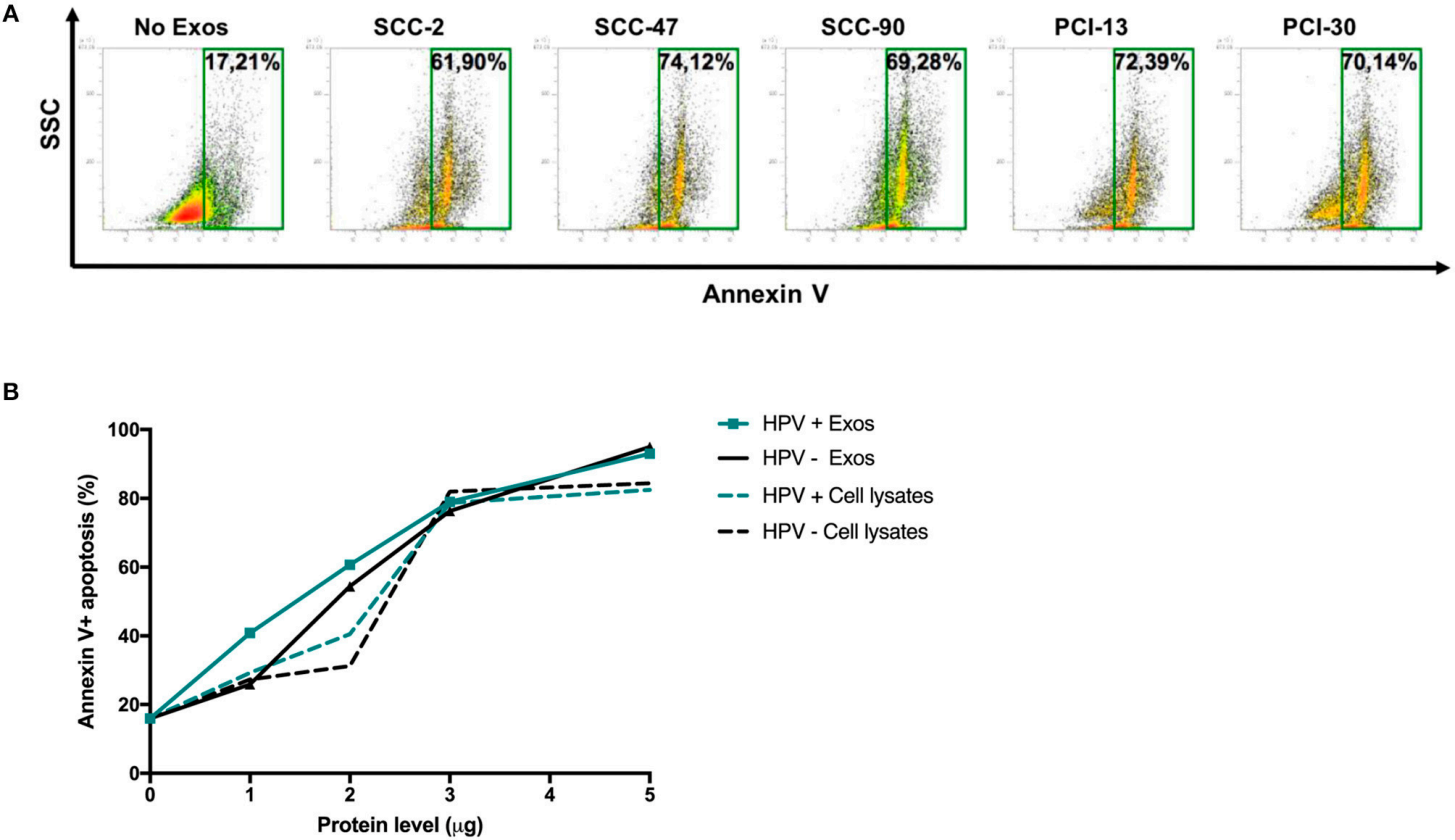
Exosome-induced apoptosis of CD8^+^ Jurkat cells. **(A)** Representative flow cytometry data for Annexin V-stained CD8^+^ Jurkat cells incubated with exosomes or PBS as control for 24 h. **(B)** Cumulative data from experiments in which CD8^+^ Jurkat cells were co-incubated with increasing protein levels of exosomes isolated from supernatants of HPV(+) or HPV(−) cell lines or with lysates of these cell lines.

Figure 5 shows that HPV(+) as well as HPV(−) exosomes mediated apoptosis of CD8^+^ Jurkat cells. While Jurkat cell apoptosis by tumor-derived exosomes was concentration dependent (Figure 4B), the percentages of cells undergoing apoptosis were not significantly different for HPV(+) and HPV(−) exosomes. Also, exosomes mediated apoptosis of Jurkat T cells somewhat more effectively than did cell lysates (Figure 4B).

**Figure 6:**
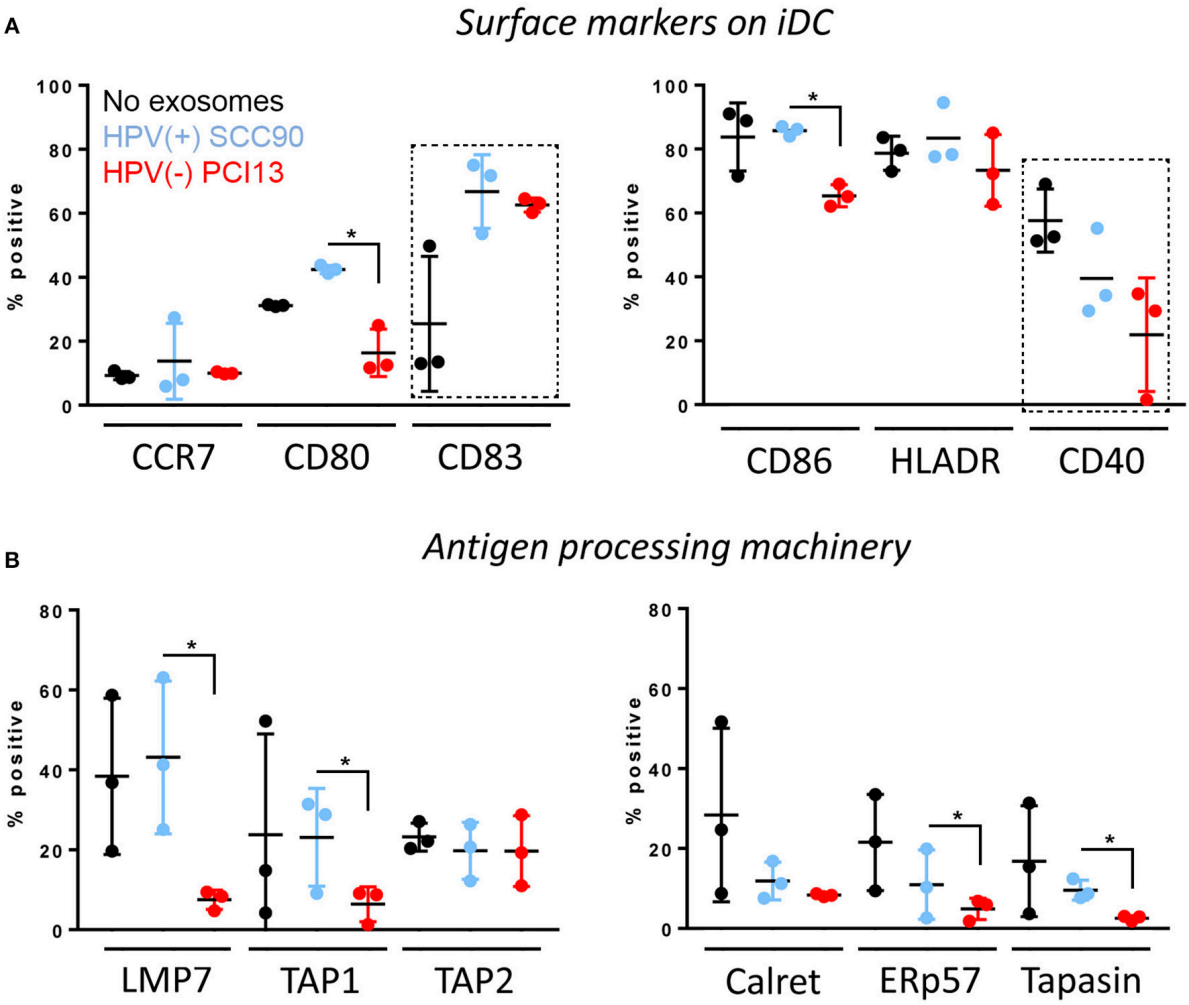
Effects of HPV(+) and HPV(−) exosomes on immature human dendritic cells (iDC). Co-incubation of HPV(+) or HPV(−) exosomes with human PBMC-derived monocytes. The exosomes were added to monocytes on days 0 and 3 of culture as described in Methods. iDC were harvested on day 6 and were studied by flow cytometry for expression levels of co-stimulatory proteins on the cell surface in **(A)** or the APM components in the cytoplasm in **(B)**. The data are from 3 experiments performed with iDCs generated from monocytes of 3 healthy donors. Stars denote significant differences between HPV(+) and HPV(−) exosomes at *p* < 0.05. Dotted squares indicate differences at *p* < 0.05 between iDC + no exosomes vs. iDC co-incubated with either HPV(+) or (HPV(−) exosomes). All other differences between HPV(+) and HPV(−) exosomes are nsd.

The HPV(+) or HPV(−) exosomes were co-incubated with monocytes isolated from normal human PBMC and undergoing differentiation into immature dendritic cells (iDC) in the presence of GMCSF and IL-4. The data shown in Figure 6A indicate that only HPV(+) exosomes up-regulated CD80 (*p* < 0.005) as well as CD83 (*p* < 0.04) expression levels on the iDC surface. In contrast, HPV(−) exosomes downregulated expression levels of CD80 (*p* < 0.05), CD86 (*p* < 0.05) and CD40 (*p* < 0.05) on the iDC surface. As these co-stimulatory proteins are necessary for mature DC to effectively signal to T-cells, the data suggests that HPV(+) exosomes promoted iDC maturation, while HPV(−) exosomes inhibited iDC maturation. Further, only HPV(−) exosomes down-regulated expression levels of the APM components LMP7 (*p* < 0.05), TAP1(*p* < 0.05), ERp57 (*p* < 0.05), and Tapasin (*p* < 0.05) in iDC (Figure 6B). These results suggest that while HPV(+) exosomes support differentiation and maturation of iDC, HPV(−) exosomes tend to impede monocyte differentiation into iDC and to down-regulate expression of selected APM components in iDC.

### Functions of exosomes isolated from plasma of HPV(+) and HPV(−) HNC patients

**Figure 7:**
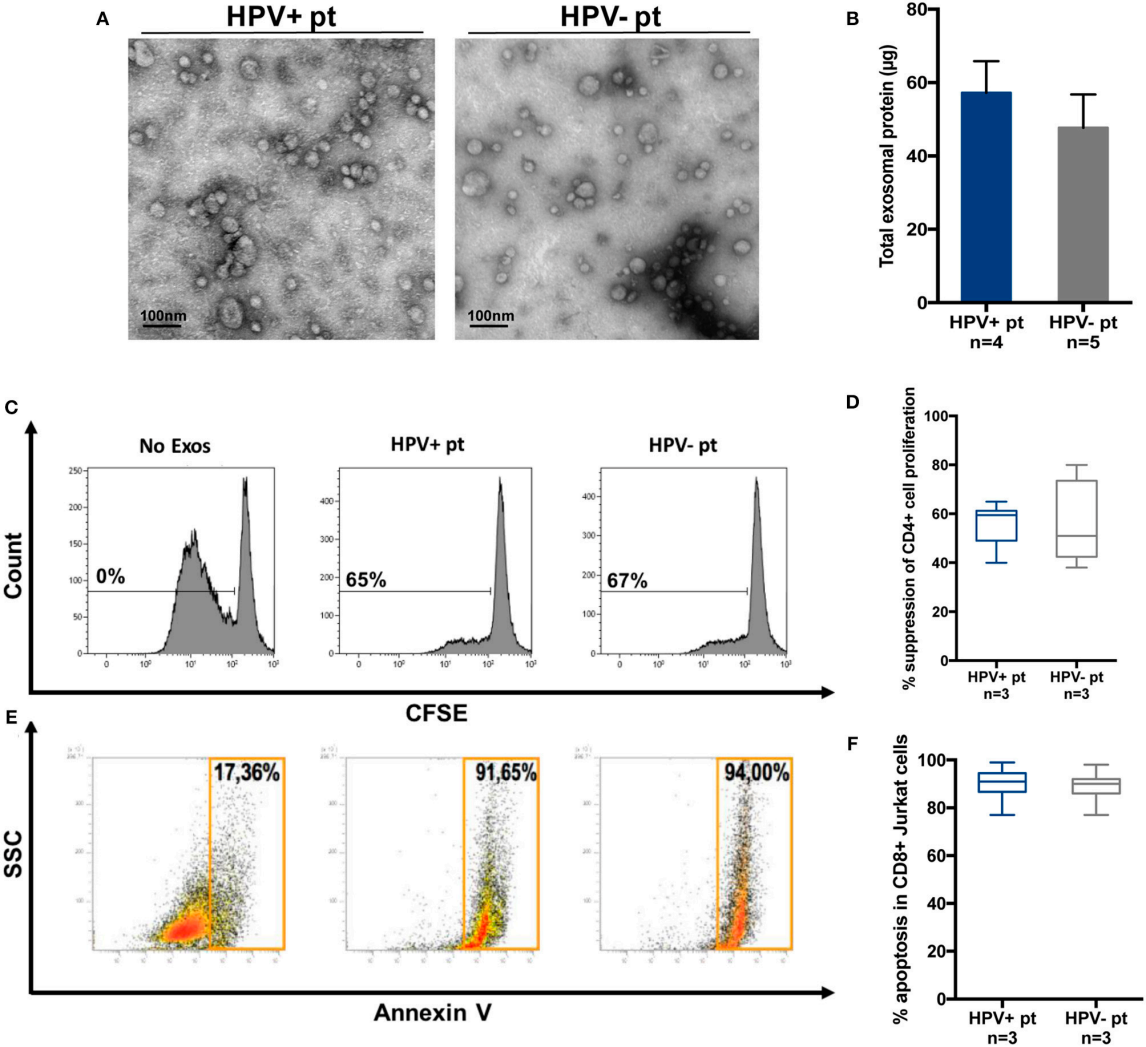
Molecular and functional characteristics of exosomes isolated from plasma of HPV(+) or HPV(−) patients. **(A)** Representative TEM images of plasma exosomes from HPV(+) and HPV(−) patients showing a diameter range of 30–100 nm. **(B)** Protein levels in exosomes of HPV(+) and HPV(−) patients. **(C,D)** Representative flow data and cumulative data for CFSE-based proliferation assays with CD4^+^ T cells co-incubated with plasma exosomes of HPV(+) or HPV(−) patients. **(E,F)** Apoptosis induction in CD8^+^ Jurkat cells co-incubated with exosomes isolated from plasma of HPV(+) or HPV(−) patients.

Exosomes were also isolated by miniSEC from plasma of a few patients with HPV(+) and HPV(−) HNC. The objective was to see whether the effects of these exosomes on functions of human immune cells were similar to those mediated by the tumor cell line-derived exosomes. Figure 7A shows that plasma-derived exosomes were similar in size and morphology to cell line-derived vesicles. HPV(+) and HPV(−) plasma contained similarly high levels of exosome proteins (Figure 7B), and similarly inhibited CD4^+^ T-cell proliferation (Figures 7C, D) or induced apoptosis of CD8^+^ Jurkat cells (Figures 7E, F).

## Discussion

This study compared molecular contents and functional characteristics of exosomes produced by HPV(+) and HPV(−) HNSCC cell lines. The rationale for this comparison was based on the premise that exosomes which originate from the endocytic compartment of the parent tumor cell carry proteins that simulate the molecular content and functions of the parent. Thus, exosomes that mimic the parent cell could serve as surrogates of HPV(+) or HPV(−) tumor cells reflecting the effects these tumor-derived exosomes exert on tissue and immune cells. Using an experimental model of exosomes produced by HPV(+) and HPV(−) cell lines, we evaluated exosome-driven reprogramming of immune cells by HPV(+) exosomes carrying E6/E7 vs. that induced by HPV(−) exosomes. We expected that molecular and immunological analyses of exosomes would uncover differences between HPV(+) vs. HPV(−) tumor cells that drive biological events culminating in distinct sensitivity of the tumors to anti-tumor therapy and ultimately outcome.

Surprisingly, despite considerable differences in the protein profiles, there were no differences in T-cell responses to HPV(+) vs. HPV(−) exosomes. Functions of CD4^+^ and CD8^+^ T cells were suppressed by these exosomes. However, DC maturation and expression of the APM components were down-regulated by HPV(−) exosomes. In contrast, HPV(+) exosomes up-regulated expression of co-stimulatory CD80 and CD83 molecules on iDCs and did not inhibit expression of the APM components. Thus, human monocyte-derived iDC and mDC responded differently to HPV(+) vs. HPV(−) exosomes, while T cell responses were equally inhibitory with both. Interestingly, we previously showed that HNSCC-derived exosomes promoted proliferation and suppressor functions of CD4^+^CD39^+^ Treg (13, 20), while they suppressed effector T cell proliferation. These previous and current findings suggest that recipient cells determine the quality of response to exosomes, possibly by regulating exosome interactions with the immune cell surface and/or internalization of exosomes. Confocal microscopy confirmed rapid uptake of tumor-derived HPV(+) and HPV(−) exosomes into DCs within 15 min of contact. In contrast, T cell did not begin exosome internalization until 12–24 h later, suggesting that prolonged initial contact with cell surface receptors drives exosome-mediated responses in T cells. We have previously reported that unlike NK cells, B cells or monocytes, T lymphocytes are reluctant to uptake exosomes (26). This study further suggests that exosome-mediated reprogramming of immune cells engages different mechanisms depending on the nature of the recipient cell. These mechanisms might include, among others, receptor-ligand signaling on the cell surface or the delivery of diverse exosome cargos to the recipient cell interior (27). T lymphocytes mainly utilize the former mechanism, while strongly phagocytic DCs undergo transcriptional alterations induced upon transfer of mRNA or miRNAs (28). HNCs are known to contain numerous miRs and to package them in exosomes (14). We have recently identified 8 miRNAs that were over-expressed in HPV(+) exosomes and 14 that were overexpressed in HPV(−) exosomes. The analysis of miRs in HPV(+) vs. HPV(−) exosomes is currently in progress. It is quite likely, however, that differential responses of DCs to HPV(+) vs. HPV(−) exosomes are mediated at the transcriptional level following internalization of exosomes by DC.

The isolated HPV(+) and HPV(−) exosomes were morphologically indistinguishable, were equally numerous and were equally well armed with immunosuppressive proteins, as previously reported for exosomes from plasma of HNC patients (13). Thus, it was not surprising that both HPV(+) and HPV(−) exosomes efficiently down-regulated functions of activated T cells. Nevertheless, we reasoned that the content of exosomes produced by virus-infected parent cells is likely to be modified compared to exosomes released by non-infected cells. We showed that exosomes produced by HPV(−) HNC cells were not only deficient in E6/E7 proteins but also in p16, survivin and cyclin D1, indicating that HPV(+) and HPV(−) exosomes have distinct protein profiles. Since the viral antigens may trigger potent immunity, we expected that exosomes produced by HPV(+) tumor cells carrying E6/E7proteins would be strongly immunostimulatory in assays with human T lymphocytes, especially since these exosomes also carried co-stimulatory OX40 and OX40L and HSP70 molecules. Instead, these exosomes consistently induced suppression or apoptosis of activated human T cells. Our recent data show that the ratio of immune suppressive/stimulatory proteins in the exosome membrane strongly impacts exosome abilities to mediate T-cell suppression (29). The presence in the HPV(+) as well as HPV(−) exosomes of FasL, LAP-TGFβ and potentially other tumor-derived immunosuppressive proteins appears to counterbalance co-stimulatory signaling, leading to surface receptor-mediated suppression of T-cell functions by tumor-derived exosomes regardless of their HPV status. Because HPV(+) and HPV(−) exosomes derived from plasma of patients who typed as HPV(+) or HPV(−) based on p16 analyses were equally efficient in their ability to mediate immune suppression or apoptosis in activated human effector T cells, we concluded that tumor-derived exosomes carrying an excess of inhibitory ligands and paucity of co-stimulatory proteins are likely to be immunosuppressive, as also previously reported (29).

Interestingly, HPV(+) but not HPV(−) exosomes stimulated *in vitro* differentiation of human monocytes into iDCs, inducing rapid maturation of monocytes to iDC. HPV(−) down-regulated expression levels of several APM components, including TAP1. The functional disparity between HPV(+) vs. HPV(−) exosomes, with only the former producing exosomes that can stimulate iDC differentiation and sustain antigen presenting capability of DCs, could perhaps explain the more effective generation of virus antigen-specific immunity in patients with HPV(+) OPSCC as recently reported by Welters et al. (30). Intra-tumoral immune cells in HPV(+) OPSCC were reported to be enriched in activated viral antigen-specific CD161+ and CD103+ T cells, DC and DC-like macrophages (30). The presence of these polarized type I immune cells among the tumor-infiltrating lymphocytes (TILs) correlated with better overall patients' survival and favorable responses to therapy. For the first time, that report linked the presence of local HPV-specific immunity detected in TILs with good prognosis in patients with HPV(+) OPSCCs (30). Nevertheless, the frequency of virus antigen-specific T cells was low, as without *ex vivo* expansion. HIV-specific T cells were detected only in a minority of patients and were unable to respond to a challenge with viral peptides. The data presented in this seminal study fit well with the perception of the TME in HNSCCs, where tumor-derived factors mediate strong and pervasive down-regulation of anti-tumor immunity as suggested by us and others (31).

In the context of the data described above, our studies emphasize a key role of exosome-mediated reprogramming of immune cells in the promotion of tumor progression. The exosomes produced by HPV(+) or HPV(−) tumors were equally immunosuppressive in *ex vivo* functional assays with human T cells, but only those from HPV(+) tumors were T-cell stimulatory. This suggests that immune activation mediated by HPV(+) exosomes might play a dominant role in anti-tumor immune responses, as previously suggested (6–9, 32) and may contribute to greater sensitivity of HPV(+) tumors to conventional oncological therapies.

## Ethics statement

This study was carried out in accordance with the recommendations of the Institutional Review Board of the University of Pittsburgh and the protocols were approved by them (IRB#960279, IRB#0403105, IRB#0506140). All subjects gave written informed consent in accordance with the Declaration of Helsinki.

## Author contributions

SoL performed experiments and drafted the manuscript. PS performed experiments, especially functional studies with T cells. M-NT performed experiments with dendritic cells and exosomes. MP was involved in exosome. characterization and results interpretation. SY performed studies of exosome uptake by T cells and DC and confocal microscopy. StL provided financial support for SoL. SF provided mAbs that are specific for APM components and help for results interpretation. TW provided conceptual and financial support, supervised experimental work, and wrote the manuscript.

### Conflict of interest statement

The authors declare that the research was conducted in the absence of any commercial or financial relationships that could be construed as a potential conflict of interest.

## Abbreviations

HPV: human papilloma virus
HNSCC: head and neck squamous cell carcinoma
OPSCC: oropharyngeal squamous cell carcinoma
HNC: head and neck cancer
TAA: tumor-associated antigens
APM: antigen processing machinery
WB: western blots
TEM: transmission electron microscopy
PBMC: peripheral blood mononuclear cells
DC: dendritic cells
TGF-β1: transforming growth factor-beta
SEC: size exclusion chromatography
TME: tumor microenvironment.
