# Supplementary material for: Molecular and Functional Profiles of Exosomes From HPV(+) and HPV(−) Head and Neck Cancer Cell Lines
Source: Front Oncol. 2018 Oct 12;8:445. doi: 10.3389/fonc.2018.00445 (PMC6194188; doi:10.3389/fonc.2018.00445)

**Supplementary Data (Expanded View)**

**Molecular and functional profiles of exosomes from HPV(+) and HPV(-) head and neck cancer cell lines**

Sonja Ludwig, Priyanka Sharma, Marie-Nicole Theodoraki, Monika Pietrowska, Saigopalakrishna S. Yerneni, Stephan Lang, Soldano Ferrone, Theresa L. Whiteside





**SupplementaryTable 1**. The table summarizes the cell line origin and viral status as well as the TNM status of the patients whose tumors were used to establish the HPV+ and HPV**-** cell lines used in this study.

**

**

**Supplementary Figure 1**. Results of qRT-PCR analysis documenting the presence of mRNAs for E6 and E7 in the HPV+ cell lines and the absence of the transcripts in the HPV**-** cell lines used as a source of exosomes in this study.

**Supplementary Figure 2.** Full Western blot images for Figure 2


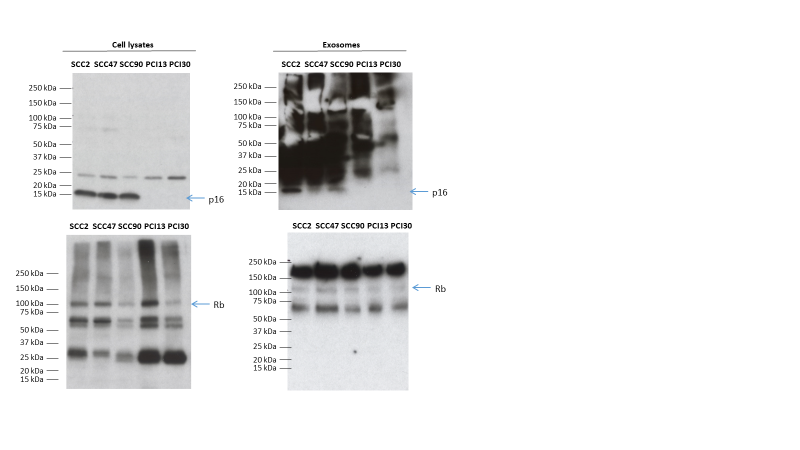


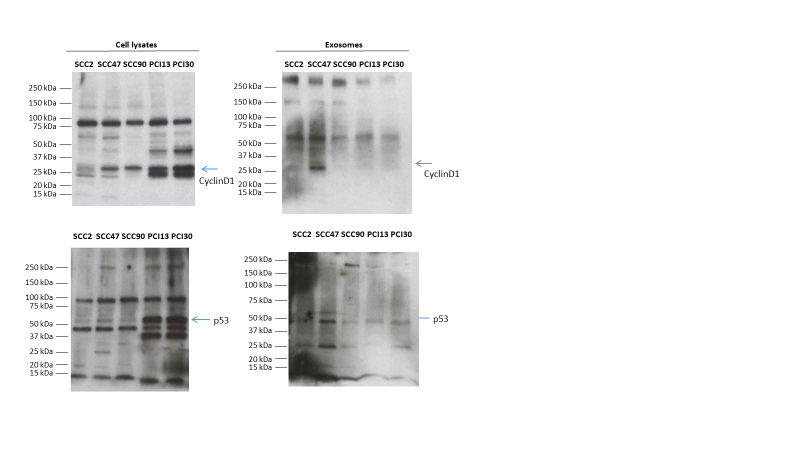


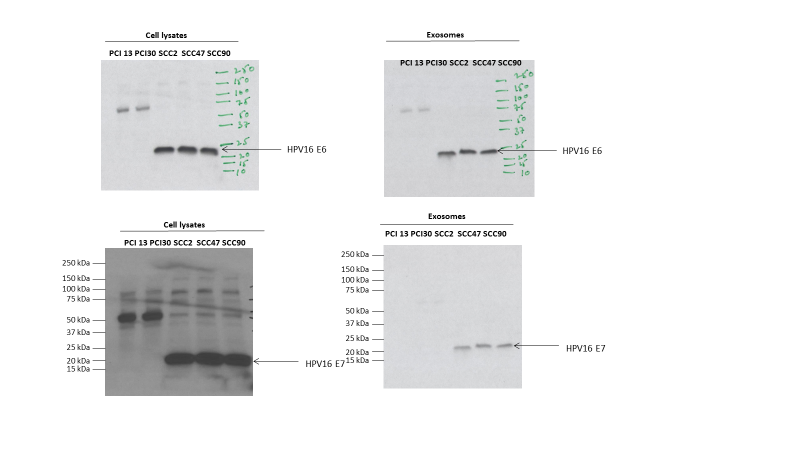

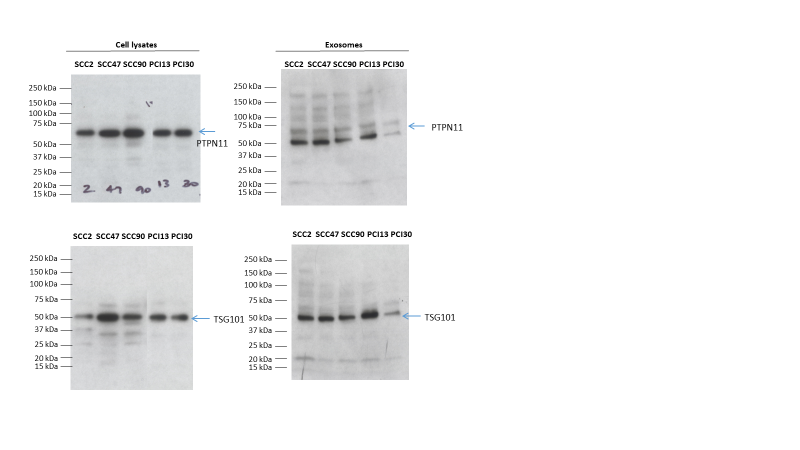


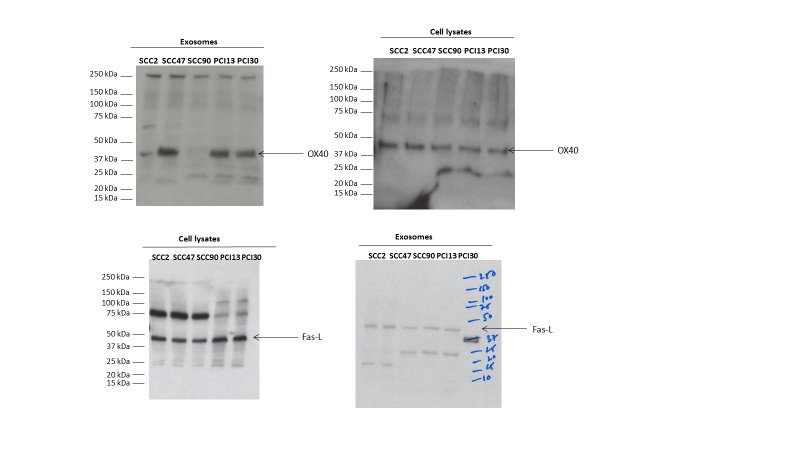

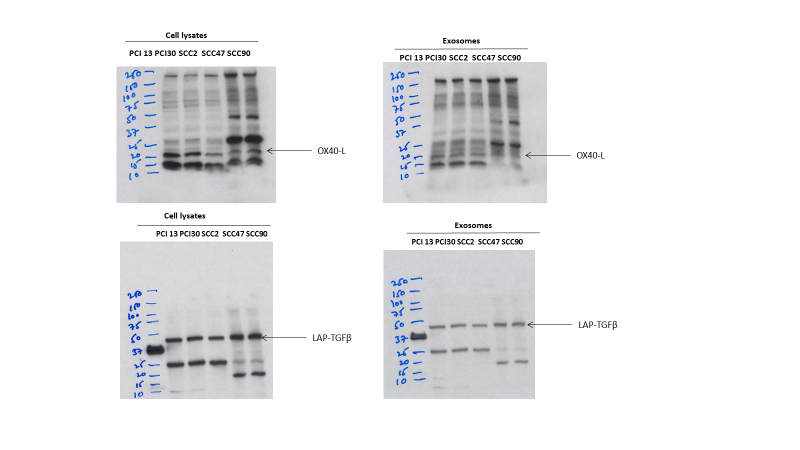


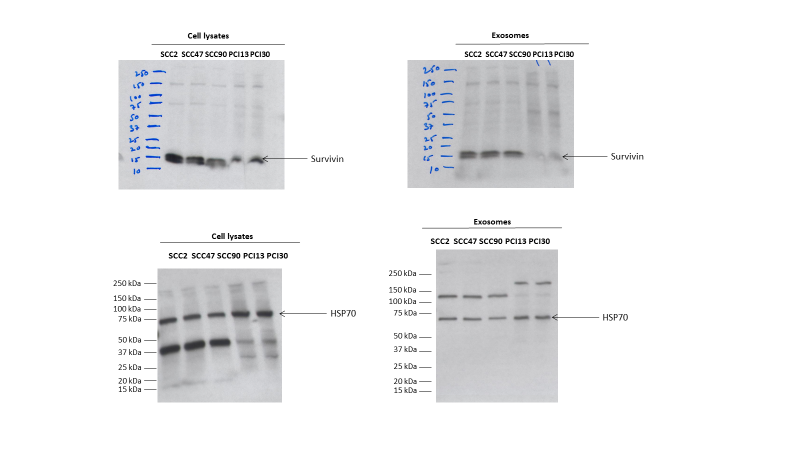


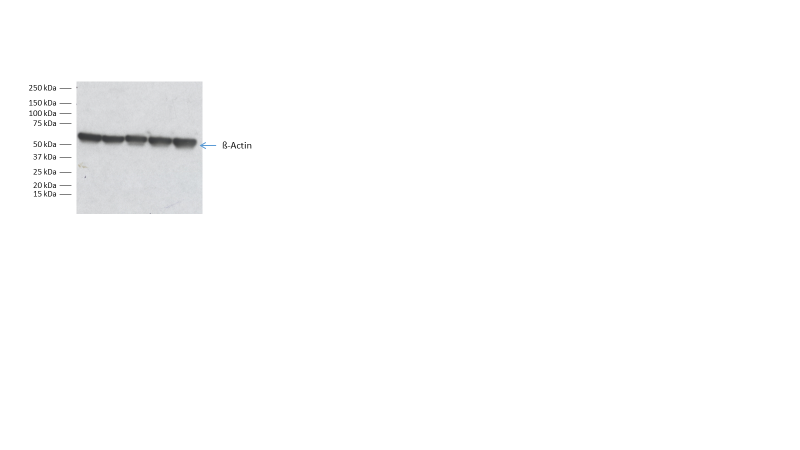

Supplement: Supplementary file 1 [file Data_Sheet_1.DOCX]
